# Relationship between motor fitness, fundamental movement skills, and quality of movement patterns in primary school children

**DOI:** 10.1371/journal.pone.0237760

**Published:** 2021-05-26

**Authors:** Hua Wu, Wichai Eungpinichpong, Hui Ruan, Xinding Zhang, Xiujuan Dong

**Affiliations:** 1 Faculty of Associated Medical Sciences, Khon Kaen University, Khon Kaen, Thailand; 2 Faculty of Physical Education, Hainan Normal University, Haikou, China; 3 Faculty of Associated Medical Sciences, PT Division of Physical Therapy, BNOJHP Research Center, Khon Kaen University, Khon Kaen, Thailand; Hochschule Trier, GERMANY

## Abstract

Seefeldt`s classic motor development pyramid model recognizes the significance of fundamental movement skills (FMS) in physical activities and proposes a “proficiency barrier” between FMS and higher-level specific sports skills during middle childhood. However, the relationship between the layers of the conceptual model has not been empirically tested. This study investigated motor fitness (MF), FMS, and quality of movement patterns (QMP) in 7–10 years old children and evaluated the relationships among them. A total of 117 children were randomly selected to take tests of MF, the Test of Gross Motor Development-2 (TGMD-2), and the Functional Movement Screen (FMS^™^). MF and FMS levels were classified according to percentile ranges. Two multiple (R×C) Chi-Square tests were applied to analyze the relationships between MF, FMS, and QMP. Post-hoc testing estimated the possibility of FMS and QMP to predict MF. The results showed that boys scored significantly higher on the object-control subtest and on the TGMD-2 compared to girls (p<0.001), while girls scored significantly higher on the FMS^™^ (*p* = 0.001). FMS score and QMP level were weakly correlated with MF (FMS: χ^2^ = 14.605, p = 0.006, Cramer`s V = 0.25; QMP: χ^2^ = 13.943, p = 0.007, Cramer`s V = 0.24). Thus, 60.5% of children with “excellent” FMS and 59.6% with “high” QMP were categorized as having a “good” MF. In contrast, only 23.1% of children with “poor” FMS and 24.3% with “low” QMP were classified as having a “good” MF. Our results confirm MF, FMS, and QMP are correlated with each other, although this relationship is weak. Further, a possible motor skill proficiency barrier exists already in children 7–10 years old. The study results support the promotion of physical activity and motor skill development in primary school children.

## Introduction

Childhood obesity and sedentary behavior are among the most serious global public health challenges of the 21st century and their prevalence in developing countries have increased at an alarming rate [1,2]. Data from the most recent national survey “2016 Physical Activity and Fitness in China—The Youth Study (PAFCTYS)”, which included 116,615 Chinese school children (9–17 years old), showed that 11.9% of Chinese school children were obese and 36.8% spent more than two hours a day on screens [3]. The physical activity and physical fitness data were also discouraging. Only 29.9% of children met the recommended guidelines for moderate-to-vigorous physical activity of at least 60 min/day [4] and only three out of 10 children met the national physical fitness standard of “excellent” or “good” [5]. It has been found that children with a competent level of motor skill performance are more likely to be physically active [6,7]. Malina et al. [8] also suggested that children with adequately developed motor skills were more likely to continue to engage in regular physical activity during adolescence. Fundamental movement skills (FMS) are considered to be the building blocks of more complex and advanced motor skills by classic theoretical motor developmental models, and early childhood is the best time to develop these skills [9–11]. Therefore, the role of FMS has increasingly attracted attention from scholars and has recently become a part of the early elementary school pedagogical approach for physical activity [12–14].

Motor fitness (MF) comprises several components of physical fitness, including strength, speed, flexibility, and agility [15], that is associated with enhanced performance in sports and the development of motor skills [16]. Previous studies [17,18] have reported an association between FMS and physical activity that increases gradually during childhood and adolescence and is mediated by physical fitness, perceived exercise ability, and obesity. Recent longitudinal studies have provided further evidence that developing FMS can have potential long-term effects on physical activity and health-related physical fitness [19] and that FMS, body fat, and cardiorespiratory fitness display reciprocal relationships [20]. However, the proficiency of FMS in school children has been found to be low [12,21–24]. Hardy and colleagues [25] reported that the prevalence of FMS mastery among primary school students was rarely above 50% during 13 years of school-based surveys. A significant increase in FMS capacity was observed in 2004, which was presumably due to changes in practice and policies to support FMS teaching in schools [25]. Thus, FMS can be developed, practiced, and consolidated over time with adequate exercise stimuli, professional instruction, and feedback [18,26].

Seefeldt [9] first presented the underlying role of FMS in life-long physical activity through a hypothetical motor developmental model. In this pyramid model, Level 1 comprises reflexes (e.g., grasp, startle) and postural reactions (e.g., equilibrium reactions) that first appear in infancy [27] and which are based on the development and maturation of the nervous system. These fundamental functions influence the basic mobility and stability of the body`s moving segments to create fundamental movement patterns (e.g., squatting, standing, walking, running) [28]. Level 2 of the model is linked to the acquisition of FMS, which starts in early childhood. Seefeldt [9] proposed a hypothetical “proficiency barrier” between the development of FMS in early childhood and the attainment of transitional motor skills (i.e., rope-skipping, breaststroke, hitting a ball) during middle childhood and adolescence (level 2 to 3). More specifically, he proposed that individuals with FMS incompetency will face problems achieving proficiency with complex motor skills and thereafter may not be able to take part in sport-specific activities (i.e., swimming, tennis, volleyball) at the top level of the model. Although Seefeldt`s hypothesis has been embraced and promoted in the field of motor development, there is still a lack of empirical testing of the relationships between the various layers [29].

While there are probable correlations between quality of movement patterns (QMP), FMS, and MF in the theoretical model, little is known about their relationship in primary school children. Stodden and colleagues [30] reported evidence for the possibility of a motor skill “proficiency barrier” for young adults, but empirical evidence of a possible motor skill “proficiency barrier” in children aged 7–10 years is still missing. To address this we first hypothesized that QMP, FMS and MF are correlated in primary school children. Second, we hypothesized that there is a motor skill proficiency barrier in primary school children.

## Materials and methods

### Subjects

The present study was conducted in June 2018 using a cross-sectional design to examine the relationship between MF, FMS and QMP in primary school children. We used Research Randomizer software to randomly assign four classes from different grades (grades 1–4) of a primary school (Haikou) in China. Researchers recruited the participants. Prior to the study, teachers sent invitations to the parents including information about the study, its risks and benefits, and an informed consent form. The exclusion criteria were: 1) students who could not attend the test because of a disease or disability and 2) students who were unwilling to take the test. This study proposal was approved by the Hainan Normal University Ethics Committee (2018HSKY-20) and conforms to the Declaration of Helsinki. Initially, 249 children (aged 7–10 years) took the MF test (Chinese National Student Physical Fitness Standard) [31] and the Test of Gross Motor Development-2 (TGMD-2) [32]. Subsequently, based on their TGMD-2 total scores, 120 children (the 10 best, 10 average, and 10 weakest [33] children from each of the four grades) were selected to perform the FMS^™^ [34]. The selection was done due to limited FMS^™^ test equipment and FMS^™^ test raters. Finally, 117 children (aged 8.9 ± 1.2) underwent all three tests. Three children did not complete all the tests and were excluded. Table 1 demonstrates the descriptive statistics on anthropometric data.

**Table 1:** Descriptive statistics for each grade group (mean ± SD).

Group	Age	Body Height	Body Mass	BMI	n (female)
**Grade 1**	7.4 ± 0.6	120.7 ± 4.6	22.9 ± 4.1	15.6 ± 2.5	28 (9)
**Grade 2**	8.2 ± 0.3	129.9 ± 4.8	27.3 ± 5.6	16.1 ± 2.5	29 (12)
**Grade 3**	9.2 ± 0.4	133.7 ± 4.9	29.6 ± 5.0	16.5 ± 2.6	30 (10)
**Grade 4**	10.2 ± 0.5	136.3 ± 6.6	31.4 ± 7.4	16.9 ± 3.2	30 (8)

### Measurement procedure

Prior to motor assessments, the participants performed a warm-up guided by physical education (PE) teachers which consisted of jogging followed by joint exercises. After familiarization (verbal instruction, visual demonstration, and performing one trial depending on test requirements) participating children completed the tests. The interval between each test was three days. The tests were conducted during normal school hours, the MF test was carried out on the track field, and the TGMD-2 and FMS^™^ tests on the enclosed tennis court. The MF test raters were trained PE teachers, and the TGMD-2 raters were graduate students with unified training through a PE expert using the TGMD-2 examiner`s manual and video. The FMS^™^ test rater was an expert with a qualification certificate in FMS^™^ [34,35]. The data were collected by statistical analysts who did not participate in the tests.

#### MF test

Speed, flexibility, coordination, and strength are the components of MF that are needed for success in athletics and lifetime sports and activities [31]. To assess the motor fitness components, we selected four items from the 2014 revised Chinese National Student Physical Fitness Standard (CNSPFS) [5,36] as our test. CNSPFS is a reliable and valid measurement to assess physical fitness in students and is part of the battery of official testing in China [37,38]. Grade 1 and 2 students completed a 50m sprint, sit-and-reach test, and timed rope-skipping as part of the test, while timed sit-ups were added to the three exercises mentioned above for students from Grades 3–4 (Table 2). Raw data were converted into scores following the scoring criteria. The final total score was weighted by test scores, where 70 was considered a perfect score [36].

**Table 2:** MF indicators and weight coefficient on CNSPFS among students of different grades.

School grade	MF indicators	Objective	Weight score
**Grade 1 and Grade 2**	50 m sprint	Speed of movement	20
	Sit and reach	lower-back/upper-thigh flexibility	30
	Timed rope-skipping	coordination	20
**Grade 3 and Grade 4**	50 m sprint	Speed of movement	20
	Sit and reach	lower-back/upper-thigh flexibility	20
	Timed rope-skipping	coordination	20
	Timed sit-ups	Abdominal strength and endurance	10

*Speed*. Speed was assessed using the 50m sprint test. The participants were advised to run 50 meters straight and as fast as possible on a smooth and clear surface. The test was performed once by each participant and the result is reported to the nearest 0.1 s.

*Flexibility*. The participants were instructed to take a sitting position with the knees fully extended and the foot placed against vertical support. They were asked to stretch out their hands as far forward as possible along with a measuring tape. Each participant performed the sit-and-reach test twice, and the best recording score was chosen (with an accuracy of 0.1 cm).

*Coordination*. The participants were instructed to perform the timed rope-skipping test once. Each participant was asked to jump for one minute in a row and they had to take off and land always on both feet. The total number of jumps was recorded.

*Muscular endurance*. The participants completed the timed sit-ups test once. They were instructed to lie in a supine position with knees bent and feet flat on the floor mat, with hands placed behind their head and fingers crossed. They elevated their trunk until the elbow touched the thigh and returned to the starting position by lowering their shoulder blades back to the mat. During the one-minute test, they were asked to do as many sit-ups as possible. The examiner counted and recorded the number of sit-ups.

#### Test of gross motor development-2 (TGMD-2)

The TGMD-2 [32] is used to assess the development of FMS in children aged 3–10 years in many countries and has high validity and reliability. In their systematic review, Rey et al. [39] concluded that the TGMD (regardless of TGMD-2/TGMD-3 version) had moderate-to-excellent internal consistency, good-to-excellent inter-and intra-rater reliability, and moderate-to-excellent test-retest reliability. For Chinese children, the TGMD-2 has been found to be reliable (inter-rater r = 0.86; internal consistency ɑ = 0.76–0.96; test-retest r = 0.87–0.95) and valid [40,41]. The TGMD-2 is comprised of a locomotor subtest (running, galloping, hopping, leaping, horizontal jumping, and sliding) and an object-control subtest (striking a stationary ball, stationary dribbling, catching, kicking, overhand throwing and underhand rolling). Each subtest has 3–5 behavior components as a mature pattern of the skill. If a child performed correctly on the behavioral component, the rater marked 1, otherwise a 0 was marked. During the test, two raters scored independently at the same time (Pearson correlation coefficient was used to evaluate inter-rater reliability within our group. r = 0.63~0.81), and the average score of two examiners was taken as the final result. The raw scores of the TGMD-2 are 48 for each of the locomotor (LM) and object-control subscales (OC), and 96 is the sum of LM and OC.

#### FMS^™^

The QMP was assessed using the FMS^™^ test [17] which includes 7 fundamental movement tests based on fundamental proprioceptive and kinesthetic awareness principles. The FMS^™^ is a reliable test (intra-rater reliability k = 0.74–1) [42] used to analyze the quality of movement patterns of athletes [43]. Although no independent study has examined the reliability and validity of FMS in school-age children [44,45], it has been used in children to access functional fitness [21] and to evaluate the relationship between QMP, children`s weight status, and physical inactivity [22,23], and their athletic performance [24]. Duncan et al. [44] found that the Kappa value showed excellent test-retest consistency in both the total composite score and the FMS subset tests (Kappa = 0.97 to 1). The test battery is comprised of 10 test items including 7 main tests: Deep Squat (DS), Hurdle Step (HS), In-line Lunge (IL), Shoulder Mobility (SM), Active Straight Leg Raise (ASLR), Trunk Stability Push-Up (TSP), and Rotary Stability (RS), alongside three clearance tests: impingement clearing test, press-up clearing test, and posterior rocking clearing test. Each test was performed 3 times, evaluated with 3–5 criteria, and scores ranging from 0 to 3 points were awarded for the correct execution criteria. Participants that scored three points at any test performance were not required to repeat the test. If the participants reported pain at any time during the testing, a score of 0 was given. In all tests except for the DS and TSP, both sides of the body were assessed separately. The lower scores of both body sides were recorded and counted into the final score. The 7 test scores were compiled into a total of 21 points.

### Statistical analysis

MF scores, TGMD-2 scores, and FMS^™^ scores were checked for normal distribution using the Kolmogorov–Smirnov test. Data are presented as group means values and standard deviation (mean ± SD). The Kruskal-Wallis H test was used to determine differences in the grade groups, and the Mann-Whitney U test was used to verify differences between the gender groups. A p-value of <0.05 was considered statistically significant. Based on a study from Stodden and colleagues [30], we classified the fitness and performance levels of the participating children for MF and QMP as “good” or “high” (≥60th percentile), “fair” or “moderate” (between the 36th and 59th percentiles), and “poor” or “low” (≤35th percentile). Thereafter, the data were analyzed by two multiple (3×3) Chi-Square tests of independence. For the first Chi-Square analysis, FMS levels (T-A, T-B, T-C) were taken as the independent variable, and MF levels (good, fair, or poor) and QMP level (high, moderate, or low) as the dependent variables. Afterward, the independent variable was changed to QMP levels and MF and FMS levels were taken as the dependent variables for the second Chi-Square analysis. According to Cohen`s study [46], an effect size V = 0.1 is considered a “small” effect size, 0.3 represents a “medium” effect size, and 0.5 a “large” effect size. Post-hoc analysis was performed based on adjusted standardized residuals to determine the statistical significance of individual cells. The adjusted standardized residuals were the difference between observed counts and expected counts, divided by the estimated standard error.

## Results

### Basic analyses

#### Age-specific analysis

As MF, FMS, and QMP are related to age [28,47], the average scores of the MF (50.40 ± 8.22), TGMD-2 (68.36 ± 8.46), and FMS^™^ (14.29 ± 2.70) tests for all 117 children were compared to averages for the different grade groups. There were no age-related differences among the TGMD-2 and FMS^™^ scores. However, the MF test scores showed significant differences between age groups (grades; p = 0.008). The average TGMD-2 score at Grade 4 was 70.13 ± 9.68. Half of the 117 children scored less than or equal to 14 in the FMS^™^ score, which is the cutoff value of injury prediction by restricted and asymmetrical fundamental movement patterns [48,49]. Table 3 presents the descriptive results of each grade group.

**Table 3:** Age-specific analyses of MF test, TGMD-2, and FMS^™^ (mean ± SD).

	MF score	Locomotors subtest score	Object-control subtest score	TGMD-2 total score	FMS^™^ score
**Grade1**	48.76 ± 9.22	34.27 ± 3.20	31.71 ± 5.58	65.98 ± 7.26	14.75 ± 3.26
**Grade2**	53.54 ± 8.45	35.41 ± 5.45	33.60 ± 4.65	69.02 ± 9.14	14.24 ± 2.86
**Grade3**	51.31 ± 7.81	33.85 ± 4.15	34.30 ± 4.61	68.15 ± 7.33	14.13 ± 2.45
**Grade 4**	47.98 ± 6.52	35.92 ± 4.93	34.22 ± 5.52	70.13 ± 9.68	14.07 ± 2.27
**Total**	50.40 ± 8.23	34.87 ± 4.54	33.49 ± 5.14	68.35 ± 8.46	14.29 ± 2.70
**Z**	11.94	4.718	4.332	3.996	1.089
***p***	0.008*	0.194	0.228	0.262	0.78

*p<0.05.

#### Gender analysis

Boys scored significantly higher on the object-control subtest score (p<0.001) and the TGMD-2 total score (p = 0.014) than girls. However, girls achieved a significantly higher average score on the FMS^™^ (p<0.001) (Table 4). Girls scored significantly more points than the boys in the DS (p<0.01), HS (p = 0.033), and ASLR (p<0.001) parts of the FMS^™^.

**Table 4:** Gender analyses of MF test, TGMD-2, and FMS^™^ (mean ± SD).

	MF score	Locomotors subtest score	Object-control subtest score	TGMD-2 total score	FMS^™^
**Boys**	49.61 ± 8.64	35.03 ± 4.52	34.75 ± 4.65	69.78 ± 8.03	12.74 ± 2.57
**Girls**	51.85 ± 7.28	34.57 ± 4.62	31.15 ± 5.25	65.72 ± 8.70	14.73 ± 2.24
**Z**	-0.1.046	-0.815	-3.541	-2.457	-4.112
***p***	0.296	0.415	0.001*	0.014*	0.001**

NOTE: TGMD-2 total score = Locomotors subtest score + Object-control subtest score.

*Significant gender difference p<0.05.

** Significant gender difference p<0.01.

### Associations between FMS competence and levels of MF and QMP

Table 5 presents the results of the tests for the association between FMS levels and MF and QMP. The first multiple 3x3 Chi-Square tested the relationship between FMS levels (T-A, T-B and T-C that corresponded to the first second and third percentile groups in the test) and MF levels (good, fair and poor which correspond to the first second and third percentiles from the MF test). No cell was expected to return a count of less than 5, and the χ^2^ (4, N = 117) = 14.605, *p* = 0.006 (p< 0.01), Cramer`s V = 0.25, which indicates a slight positive correlation between FMS levels and MF. It was noted that 60.5% of the children were classified as T-A having “good” (≥ 60th percentile) MF levels, while only 15.8% of them had “poor” (≤ 35th percentile) MF levels. When viewed from the lesser skilled FMS participants, 53.8% in the T-C group demonstrated as having “poor” MF levels.

**Table 5:** Chi-Square Cross-Tabulations indexes for FMS levels × MF levels and FMS levels × FMS^™^ levels.

	MF index levels			Total	FMS^™^ index levels			Total
	Good	Fair	Poor		High	Moderate	Low	
T-A count	23	9	6	38	23	9	6	38
Expected count	16.2	8.8	13.0	38	15.3	10.7	12	38
% within	60.5%	23.7%	15.8%	100%	60.5%	23.7%	15.8%	100%
Adj.Std.Res.	(2.7)*	(0.1)	(-2.9)*		(3.1) *	(-0.8)	(-2.6)*	
T-B count	18	9	13	40	11	12	17	40
Expected count	17.1	9.2	13.7	40	16.1	11.3	12.6	40
% within	45%	22.5%	32.5%	100%	27.5%	30%	42.5%	100%
Adj.Std.Res.	(0.4)	(-0.1)	(-0.3)		(-2.0)*	(0.3)	(1.8)	
T-C count	9	9	21	39	13	12	14	39
Expected count	16.7	9	13.3	39	15.7	11.0	12.3	39
% within	23.1%	23.1%	53.8%	100%	33.3%	30.8%	35.9%	100%
Adj.Std.Res.	(-3)*	(0)	(3.2)*		(-1.1)	(0.4)	(0.7)	
Total count	50	27	40	117	47	33	37	117
Expected count	50	27	40	117	47	33	37	117
% within	42.7%	23.1%	34.2%	100%	40.2%	28.2%	31.6%	100%

Note:

* Adjusted standard residuals (Adj.Std.Res.) statistically significant different probability than expected.

The second multiple 3×3 Chi-Square tested the relationship between FMS levels and QMP, according to grouping from the FMS^™^ test into high, moderate and low percentiles. Again, no cell was expected to return a count of less than 5, and the χ^2^ (4, N = 117) = 11.118, p = 0.025 (p< 0.05), Cramer`s V = 0.22 indicated that there was a slight positive correlation between FMS levels and QMP. A similar phenomenon occurred in 60.5% of children who were sorted as the T-A group, which presented “high” FMS^™^ scores, and 15.8% showed “low” scores. However, 33.3% of children in the T-C group exhibited a “high” FMS^™^ score, while 35.9% got a “low” FMS^™^ score (Table 5).

### Associations between QMP levels and levels of MF and FMS

The third Chi-Square test (QMP levels × FMS levels) analysis, indicated a similar trend. Through post-hoc testing shown in Table 6, we can see that 48.9% of children who classified as the high FMS^™^ group, scored “well” on the TGMD-2 (T-A group); 27.7% showed “low” TGMD-2 scores (T-C group). Likewise, only 16.2% of the “low” FMS^™^ group, performed adequately in the TGMD-2 (T-A group). In the fourth Chi-Square analysis (association between FMS^™^ levels and MF levels), no cell should have counted less than 5, χ^2^ = 13.943, *p* = 0.007< 0.01, Cramer`s V = 0.24. The Chi-Square analysis indicated that 59.6% of “high” FMS^™^ students got a “good” score on the MF test, while 54.1% were classified as “low” FMS^™^ students, who presented a “poor” MF performance.

**Table 6:** Chi-Square Cross-Tabulations indexes for FMS^™^ levels ×MF levels and FMS^™^ levels ×FMS levels.

	MF index levels			Total	FMS index levels			Total
	Good	Fair	Poor		T-A	T-B	T-C	
High count	28	9	10	47	23	11	13	47
Expected count	20.1	10.8	16.1	47	15.3	16.1	15.7	47
% within	59.6%	19.1%	21.3%	100%	48.9%	23.4%	27.7%	100%
Adj.Std.Res.	(3.0) *	(-0.8)	(-2.4)*		(3.1) *	(-2.0)	(-1.1)	
Moderate count	13	10	10	33	9	12	12	33
Expected count	14.1	7.6	11.3	33	10.7	11.3	11.0	33
% within	39.4%	30.3%	30.3%	100%	27.3%	36.4%	36.4%	100%
Adj.Std.Res.	(-0.5)	(1.2)	(-0.6)		(-0.8)	(0.3)	(0.4)	
low count	9	8	20	37	6	17	14	37
Expected count	15.8	8.5	12.6	37	12.0	12.6	12.3	37
% within	24.3%	21.6%	54.1%	100%	16.2%	45.9%	37.8%	100%
Adj.Std.Res.	(-2.7) *	(-0.3)	(3.1) *		(-2.6)*	(1.8)	(0.7)	
Total count	50	27	40	117	38	40	39	117
Expected count	50	27	40	117	38	40	39	117
% within	42.7%	23.1%	34.2%	100%	32.5%	34.2%	33.3%	100%

Note:

* Adjusted standard residuals (Adj. Std. Res.) statistically significant different probability than expected.

## Discussion

Our results are consistent with previous research that the TGMD-2 and FMS^™^ are process-oriented tests that showed a gender difference in the quality of motor skills and motor patterns, which is consistent with the general phenomenon that scores are mainly influenced by biological and environmental factors [50,51]. In contrast, the MF test is a product-oriented test that may have been influenced by factors such as the small sample size and variations in individual student`s physiological and psychological situations. In our study, MF scores showed significant fluctuation in age-groups, which may affect the relationship of MF with FMS and QMP. There were significant low positive correlations between FMS and MF, which is partly consistent with Stodden et al. [30], who reported a low to moderate association between motor skill competence and health-related fitness in young adults. It consistents with previous research showing that FMS proficiency is positively associated with cardiorespiratory fitness [52,53], balance [54] physical activity [55,56], and weight [57,58] in primary school children. These correlations are consistent with Stodden`s conceptual model [18] and are broadly in line with previous reports of the positive correlation of QMP with physical fitness [44,59]. QMP represents flexibility and stability, which are the first level of functional movement, and according to the “Performance Pyramid Model” of Cook and colleagues [28], this first level supports other levels, like endurance, strength, speed, and power. Yıldız et al. reported that QMP has a significant association with agility and acceleration in child tennis players [60] and with flexibility and vertical jumping in elite Karate athletes [61]. While there is no evidence reporting the relationship between QMP and FMS, Lester et al. [62] combined QMP and FMS assessment in primary children but did not discuss any association.

In accordance with our first hypothesis, when we compare our results to other studies, it must be noted that the weak correlation we identified suggests that QMP and FMS are not strong predictors of MF in the Seefeldt`s model [9]. If the fluctuation in MF scores could be reduced by increasing the sample size or by choosing an alternative test to reflect physical fitness, the correlations we observed may be strengthened. Therefore, we ascribe MF to be affected by a combination of factors, not only motor skills, but also coordination, and physiological and psychological developments. Tompsett et al.`s review indicated that FMS intervention improved FMS proficiency, but had little effect on strength or flexibility among children and adolescents aged 5–18 years [63]. In addition, QMP has previously been considered to be ineffective in predicting performance in recreational athletes [64] as it does not assess strength [65].

The second aim of this study was to investigate whether there is a motor skill “proficiency barrier” in primary school children. Our data may indicate the existence of this “proficiency barrier”. For example, children with excellent FMS tended to score well on MF tests and those with low FMS tended to score lower on MF tests. Similarly, children in the lowest FMS group were more likely to score “poor” on the MF. This evidence reflects the importance of FMS in motor development, as FMS provides the foundation for various physical activities in the pyramidal hierarchical model of motor development [9]. Children that cannot master a wider range of FMS may have acquired a “proficiency barrier” to develop motor skills and promote physical fitness [9]. This opens the possibility of using moderate-to-vigorous physical activity guidelines for children with low FMS to provide them with the chance to develop their motor capacity [66].

A limitation of this study is that TGMD-2 test norms are lacking in the Chinese children population. We chose the top, middle, and last ten TGMD-2 scores to take the FMS^™^ test, which may have contributed to a gender imbalance in the sample. MF level is only partly reflected in physical fitness and performance in primary school children. The small sample size and external (environmental) and internal (physical and cognitive development) differences are also likely to influence FMS, MF, and QMP scores, which would affect the strength of correlations between them. Due to the cross-sectional design of this study, we could not conclude a causality in the associations. Rather, we explored a method and provided evidence to discuss how important these motor skills were for children. In the future, the cause-and-effect relationship and mechanism between FMS and other indicators related to children, especially in early childhood, should be verified. Finally, we cannot ignore the social-ecological correlates (e.g., individual physical, psychological, and social-cultural factors, and the educational environment) that may affect and restrict motor development [67–70].

Our cross-sectional data provide indirect evidence to verify the relationship among QMP, FMS, and MF in Seefeldt`s pyramid model. Moreover, the weak relationship we found between them suggests the main emphasis of FMS development exercises in primary children`s physical education, attention should be paid to consolidating QMP and enhancing MF. This suggestion is in line with the Youth Physical Development Model. This model postulates that FMS should be part of any strength and conditioning programs at all ages of athletes and that most fitness components are trainable throughout childhood with individualization according to the developmental stage [71]. Our study may also support our understanding of a possible motor proficiency barrier that influences children`s participation in physical activity. The ability to integrate a wide range of motor skills is required by continued participation in games or sports in the future [72]. This ability could be built on a wide range of fundamental movement skills, neuromuscular coordinated control, and a certain level of physical fitness.

In summary, this study has provided empirical evidence to support Seefeldt`s classical pyramid model of the relationship between QMP, FMS and MF, and the existence of a motor proficiency barrier related to MF level in children 7–10 years old. MF proficiency is closely associated with health and well-being, physical activity, and physical performance. Our data showed children with low FMS had more chance to have “poor” MF, suggesting that the development of FMS should be emphasized in primary school students, especially if their proficiency is generally low. Furthermore, we noticed a weak correlation between QMP, FMS, and MF in primary school students, and thus QMP and FMS level could not predict MF level. Therefore, while developing FMS in primary school children, some of the physical qualities are trainable as well during this period and need to be based on good QMP. Finally, it is crucially important for strengthening training to follow the principles of individualization and age development stage.
